# Comparative Proteomic Analysis of Lysine Acetylation in Fish CIK Cells Infected with Aquareovirus

**DOI:** 10.3390/ijms18112419

**Published:** 2017-11-14

**Authors:** Hong Guo, Jie Zhang, Yaping Wang, Chen Bu, Yanyan Zhou, Qin Fang

**Affiliations:** 1 State Key Laboratory of Virology, Wuhan Institute of Virology, Chinese Academy of Sciences, Wuhan 430071, China; guohong@wh.iov.cn (H.G.); zhangj@wh.iov.cn (J.Z.); 2 State Key Laboratory of Freshwater Ecology and Biotechnology, Institute of Hydrobiology, Chinese Academy of Sciences, Wuhan 430072, China; wangyp@ihb.ac.cn; 3 Jingjie PTM BioLab (Hangzhou) Co., Ltd., Hangzhou 310018, China; chen_bu@ptm-biolab.com (C.B.); yanyan_zhou@ptm-biolab.com (Y.Z.)

**Keywords:** comparative proteomics, *Ctenopharyngodon idella* kidney cells, aquareovirus infection, lysine acetylation

## Abstract

Grass carp (*Ctenopharyngodon idellus*) is an important worldwide commercial freshwater culture species. However, grass carp reovirus (GCRV) causes serious hemorrhagic disease in fingerlings and yearlings of fishes. To understand the molecular pathogenesis of host cells during GCRV infection, intensive proteomic quantification analysis of lysine acetylation in *Ctenopharyngodon idella* kidney (CIK) cells was performed. Using dimethylation labeling-based quantitative proteomics, 832 acetylated proteins with 1391 lysine acetylation sites were identified in response to GCRV infection, among which 792 proteins with 1323 sites were quantifiable. Bioinformatics analysis showed that differentially expressed lysine acetylated proteins are involved in diverse cellular processes and associated with multifarious functions, suggesting that extensive intracellular activities were changed upon viral infection. In addition, extensive alterations on host–protein interactions at the lysine acetylation level were also detected. Further biological experiments showed that the histone deacetylases (HDAC) inhibitor suberoylanilide hydroxamic acid (SAHA) could significantly suppress the GCRV replication. To our knowledge, this is the first to reveal the proteome-wide changes in host cell acetylome with aquatic virus infection. The results provided in this study laid a basis for further understanding the host response to aquareovirus infection in the post-translational modification aspect by regulating cell lysine acetylation conducive to viral replication.

## 1. Introduction

The grass carp (*Ctenopharyngodon idellus*) is an important fresh water fish in aquaculture species that has great commercial value and a worldwide distribution. Fingerlings and yearlings are predisposed to a hemorrhagic disease caused by the grass carp reovirus (GCRV), which is a double-stranded RNA virus belonging to the genus *Aquareovirus* in the family *Reoviridae* [1]. Aquareoviruses mainly infect aquatic animals such as bony fish and shellfish isolated from both sea and freshwater origins. These viruses replicate in the cytoplasm of the infected cells and hijack the host cellular machinery for generation of progeny viruses. Although most identified aquareoviruses are of low pathogenicity in breeding aquatics by routine diagnosis, GCRV can provoke severe epidemic hemorrhagic disease and pancreatitis in aquatic animals. The typical pathogenic symptom is hemorrhages in liver, kidney, gills, enteritis, and skeletal muscles, which represent a serious threat to fish breeding [2]. In addition, GCRV replicates well in the *Ctenopharyngodon idellus* kidney (CIK) cell line at 25–30 °C and produces a typical cytopathic effect consisting of large syncytia in its sensitive cells [3,4]. Previous studies on GCRV are mainly focused on viral biological and biochemical properties as well as viral three-dimensional structure based functions [5,6,7,8]. However, detailed biochemical events of biological and metabolic processes of host cells upon aquareovirus infection are poorly understood. GCRV has been recognized to be the most pathogenic among the isolated aquareoviruses [9], and it can serve as a desirable model for studying the molecular mechanism and pathogenesis of host cells in response to virus infection.

Protein post-translational modifications (PTMs) play significant roles in affecting proteins function by modulating protein activity, cellular location and protein–protein interaction. Protein acetylation on the lysine residue is a highly conserved PTM, initially discovered on histones half a century ago [10]. Lysine acetylation of host cells is reversible and dynamic due to the regulation of lysine acetyltransferases and lysine deacetylases. Additionally, the acetylation and deacetylation on the lysine residue can also function as molecular switches to initiate or inhibit protein activities or downstream events [11,12]. Lysine acetylation has been found in diverse species ranging from bacteria to humans [13], and has been reported to participate in many cellular processes including apoptosis [14,15], cell metabolism [16], cytoskeleton dynamics [17], autophagy [18], immune response [19] and so on. In recent years, with the developments in proteomic technologies and the mass spectrometry, global lysine acetylomes have been determined in a lot of species. These acetylomes have greatly increased the knowledge of lysine acetylated proteins and expanded the function diversity of lysine acetylation.

Protein acetylation also plays a critical role in host cells during viral replication. The histone deacetylases (HDACs) were reported to participate in regulation of the replication and pathogenesis of numerous human viruses including hepatitis B and C, human immunodeficiency virus (HIV), human papillomavirus (HPV), respiratory syncytial virus (RSV) and herpes viruses (reviewed in [20]). The HDAC inhibitors could significantly inhibit the RSV and hepatitis C virus (HCV) replication [21,22]. Meanwhile, the histone acetyltransferases (HATs) inhibitor C646 could suppress the influenza virus replication [23]. The human pathogen borna disease virus has been found to impact histone lysine acetylation and modify the acetylome of infected cells towards higher energy and transporter levels [24,25]. In contrast to these studies focused on human disease induced by viruses or bacteria, little is known about protein acetylation in aquatic species. Recently, the lysine acetylome has been investigated in zebrafish embryos [26], Daphnia [27], and also in the pathogenic bacterium *Spiroplasma eriocheiris* [28]. To date, there is no report on the cellular responses of the lysine acetylome in response to viral infection in aquatic species.

In the present study, the proteomic quantification analysis of the acetylome of fish CIK cells towards aquareovirus infection in combination with comprehensive bioinformatics analysis was performed. We identified 1391 lysine acetylation sites in 832 proteins which are involved in a variety of biological functions, diverse cellular processes and distributed in multiple subcellular compartments. To our knowledge, this is the first extensive dataset of lysine acetylation in fish cells infected by aquatic virus.

## 2. Results

### 2.1. Quantitative Analysis of Lysine Acetylation in CIK Cells in Response to GCRV Infection

**Figure 1:**
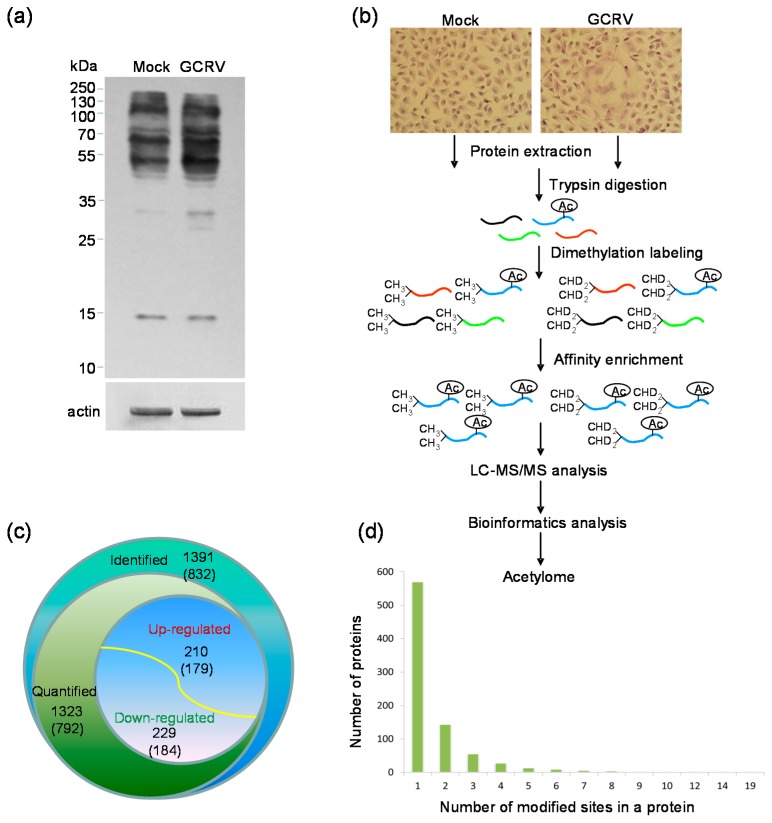
Proteome-wide identification of lysine acetylation sites and proteins in CIK cells in response to grass carp reovirus (GCRV) infection. (**a**) Lysine acetylation in GCRV infected or mock-infected cells as analyzed by Western blotting, β-actin was used as loading control; (**b**) Experimental strategy used to identify and quantify acetylated lysine sites in CIK cells in response to GCRV infection; (**c**) Number of identified and quantified lysine acetylated sites and proteins. The up-regulated and down-regulated sites and proteins were also indicated. The number of proteins was shown in brackets; (**d**) Distribution of acetylated proteins based on their number of lysine acetylation sites.

To understand whether the lysine acetylation level of cell proteins was influenced by GCRV infection, Western blotting analyses were performed with infected or mock-infected cells using the pan anti-acetyllysine and anti-β-actin antibodies. As shown in Figure 1a, compared to the protein profile in mock infected cells, both enhanced and reduced protein bands were detected at their lysine acetylation level under the same expression level of β-actin, suggesting that the lysine acetylome of host proteins were changed in response to GCRV infection. Then, we combined dimethylation labeling, HPLC fractionation, affinity enrichment and high-resolution mass spectrometry-based quantitative proteomics to comparatively quantify the detailed changes of lysine acetylome towards GCRV infection in CIK cells. The scheme of the experimental workflow is illustrated in Figure 1b. The MS data validation is shown in Figure S1. The distribution of mass error was near zero and most of them were less than 5 PPM (Figure S1a), indicating the mass accuracy of the MS data fit the requirement. In addition, the most identified peptides were distributed in the length between 7 and 19 (Figure S1b), which agree with the property of tryptic peptides. Further quantitative result showed that 1391 lysine acetylation sites in 832 proteins were identified; of which 1323 sites in 792 proteins were quantifiable (Table S1). In addition to histone proteins that were initially found to be lysine acetylated in previous report [10], the identified proteins were predominantly non-histone proteins. Among these acetylated proteins, 210 sites in 179 proteins displayed a greater than or equal to 1.5-fold increased expression and 229 sites in 184 proteins displayed a lesser than or equal to 1.5-fold decreased expression in response to GCRV infection (Figure 1c).

To evaluate the distribution of the identified lysine acetylation sites, we further calculated the modified site number in each acetylated protein (Figure 1d). Single lysine acetylation site was found in more than two-thirds (68.5%, 570 proteins) of the identified proteins. The other proteins contain different numbers of acetylation sites from 2 to 19. The cytoplasmic intermediate filament protein keratins (KRTs) KRT8 and KRT18 were both found to be altered at multiple sites, 12 sites with KRT8 and 7 sites with KRT18. A multifunctional cytoskeletal linker protein plectin, which is involved in establishment and dynamic modulation of the cytoskeletal network, was found to be changed at 14 sites. Protein disulfide isomerase family A member 3 (PDIA3), also known as ERp57 or GRP58, was modified at 9 sites. These results indicate that GCRV infection induced wide changes in host cellular proteins at lysine acetylation level.

### 2.2. Motif Analysis of the Identified Lysine Acetylation Sites and Subcellular Localization of the Differentially Expressed Lysine Acetylated Proteins

**Figure 2:**
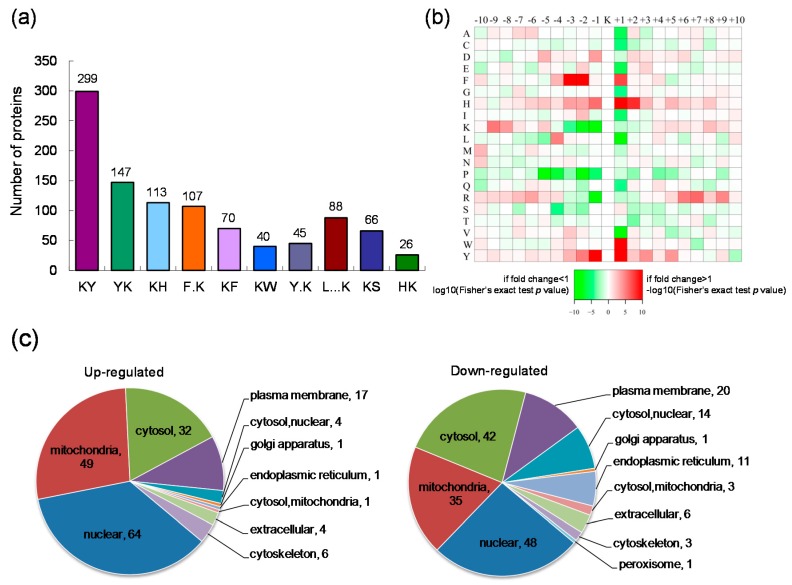
Motif analysis of the identified lysine acetylation sites and subcellular localization of the differentially expressed lysine acetylated proteins. (**a**) The number of identified proteins containing indicated lysine (K) acetylated motifs; (**b**) Heat map of the amino acid compositions around the acetylated lysine site (10 amino acids upstream and downstream of the K site). Red indicates abundant and green indicates least abundant; (**c**) The subcellular distribution of the up-regulated or down-regulated proteins.

To address the characteristics of the lysine acetylation sites changed by GCRV infection and the model of the consensus sequence motif around the sites, the lysine acetylation sequence motif analyses were performed using the Motif-x algorithm. The occupancy frequency of amino acids surrounding the acetylated lysine sites was calculated. Substantial bias in amino acid distribution around the lysine acetylation sites was observed from position −10 to +10. Ten statistically significantly enriched lysine acetylation site motifs were defined for 1001 unique sites, which accounted for 71.96% of the sites identified. As shown in Figure 2a, there is an enrichment of amino acid Y in the position +1 (29.9%), followed by the position −1 (14.7%).

To evaluate if the amino acids flanking the targeted lysine exhibit bias toward a certain motif and the significant enrichment of specific amino acids, heat maps were generated for each lysine acetylation motif. As shown in Figure 2b, the lysine acetylation of the identified proteins generally occurred in regions with a significant enrichment for tyrosine (Y), tryptophan (W), histidine (H) and phenylalanine (F) at position +1, tyrosine (Y) at position −1 and phenylalanine (F) at positions −2 and −3. The lysine acetylation also showed an absence of valine (V) and leucine (L) at position +1, lysine (K) at positions −1 and −2, arginine (R) at position −1, proline (P) at positions −2 and −5.

To address the cellular distribution properties of the quantified lysine acetylated proteins changed by GCRV infection, the predictions of subcellular localization were performed. Results showed that these lysine acetylated proteins were located in diverse cellular structures including nuclear, mitochondria, cytosol, plasma membrane, endoplasmic reticulum and so on (Figure 2c). The up-regulated or down-regulated acetylated proteins both distributed predominantly in the nuclear, followed by the mitochondria and the cytosol. In addition to up- or down-regulated plasma, membrane proteins were also highly represented in the acetylome (Figure 2c).

### 2.3. Gene Ontology (GO) Functional Classification and GO Enrichment-Based Clustering Analysis of the Differentially Expressed Lysine Acetylated Proteins

**Figure 3:**
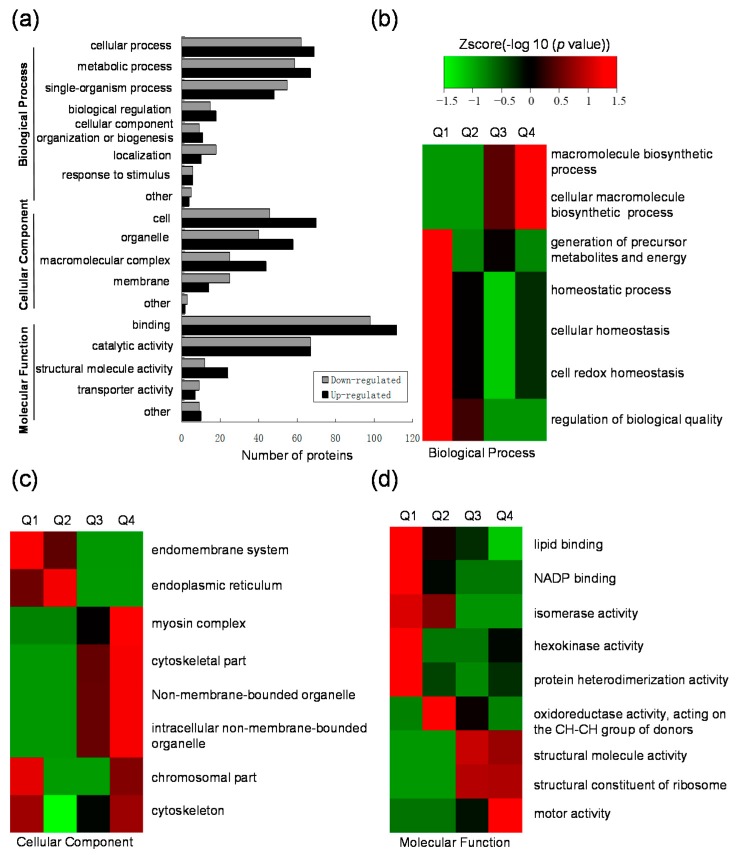
GO functional classification and GO enrichment-based clustering analysis for the differentially expressed lysine acetylated proteins. (**a**) GO functional classification of the up-regulated or down-regulated acetylation proteins based on gene ontology; (**b**–**d**) GO enrichment-based clustering analysis of the differentially expressed lysine acetylated proteins; (**b**) Biological process analysis; (**c**) Cellular component analysis; (**d**) Molecular function analysis.

To understand the nature of the differentially expressed acetylated proteins, GO functional classification analysis was performed and results are presented in Figure 3a. The results for biological process category show that proteins associated with cellular process and metabolic process form the largest and the second largest protein groups of the up- and down-regulated acetylated proteins, respectively. Another large protein group in this category were proteins involved in single-organism process. For the cellular component category, most of the differentially expressed proteins belonged to cell and organelle, followed by the macromolecular complex. In addition, proteins associated with membrane were also highly represented in acetylated proteins. The largest group in terms of molecular function was composed of binding proteins, and the second largest group in this term was proteins with catalytic activity.

To better understand the preferred functional characteristics for the differentially expressed acetylated proteins in response to GCRV infection, GO enrichment based clustering analyses were performed by dividing all quantified proteins into four quantiles according to the H/L ratios: Q1 (0~0.67), Q2 (0.67~0.77), Q3 (1.3~1.5), Q4 (>1.5). In the biological process category, the process related with macromolecule biosynthetic was found to be significantly enriched in Q4, and the processes related to metabolites and energy were highly enriched in Q1. Besides, proteins related to homeostasis such as cellular homeostasis and cell redox homeostasis processes were also highly enriched in Q1 with down-regulated lysine acetylation (Figure 3b). In the cellular component category, the up-regulated proteins were highly enriched in the myosin complex, followed by the non-membrane-bounded organelle and the cytoskeletal part, while the down-regulated proteins were enriched in endomembrane system and chromosomal part (Figure 3c). Clustering analysis based on molecular function showed that the proteins with NADP binding activity, hexokinase activity and protein heterodimerization activity were enriched in Q1 (Figure 3d). In addition, the up-regulated lysine acetylation proteins were highly enriched in motor activity, which is consistent with the over-represented myosin complex in the cellular component category. Taken together, these results indicate that the differentially expressed acetylated proteins were involved in diverse biological processes and multifarious functions.

### 2.4. Protein Domain Based Enrichment and Clustering Analysis of the Differentially Expressed Lysine Acetylated Proteins

**Figure 4:**
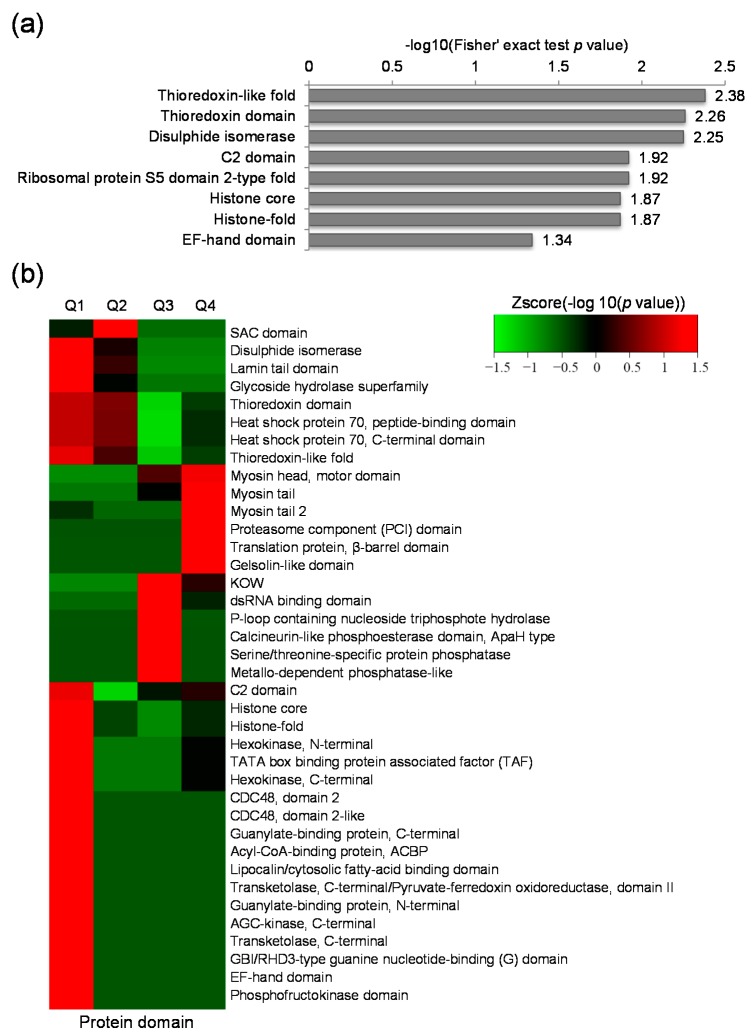
Protein domain analysis of the differentially expressed lysine acetylated proteins. (**a**) Protein domain enrichment analysis; (**b**) Protein domain based clustering analysis.

Protein domain structure is a conserved part of a given protein and can function independently of the rest part of the protein. To address the domain features of the lysine acetylated proteins altered by GCRV infection, domain annotation and enrichment analysis were performed. Results showed that thioredoxin-like fold domain, thioredoxin domain, disulphide isomerase domain were highly enriched (Figure 4a). Further clustering analysis indicated that protein domains involved in myosin tail, myosin head (motor domain), proteasome component (PCI) domain, translation protein (β-barrel domain) and gelsolin-like domain were highly enriched in up-regulated proteins (Figure 4b). The PCI domain was also significantly enriched in Q4, however, its function remains unknown. In the down-regulated proteins, various domains, such as histone core, histone-fold, glycoside hydrolase superfamily, CDC48 domain 2, CDC48 domain 2-like, guanylate-binding protein (C-terminal), acyl-CoA-binding protein, and so on were found highly enriched (Figure 4b). These data indicate that proteins with many kinds of domain features were changed in response to GCRV infection.

### 2.5. Kyoto Encyclopedia of Genes and Genomes (KEGG) Pathway Analysis of the Differentially Expressed Lysine Acetylated Proteins

**Figure 5:**
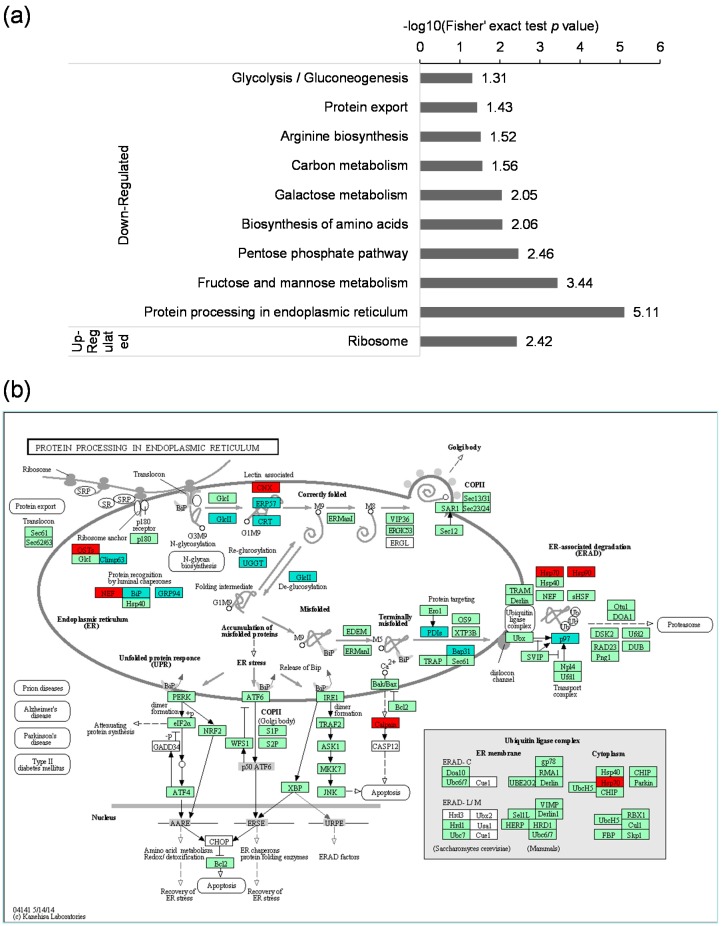
KEGG pathway analysis of the differentially expressed lysine acetylated proteins. (**a**) KEGG pathway-based enrichment analysis of up-regulated and down-regulated proteins; (**b**) The pathway obtained from KEGG pathway enrichment analysis. Changes in the lysine acetylation level of proteins in the protein processing in ER pathway in GCRV infected cells compared to mock-infected cells were indicated. The proteins in dark green are down-regulated, and the proteins in red are up-regulated. The proteins in light green are expressed at background level.

To identify pathways regulated by GCRV infection, KEGG pathway based enrichment analysis of the differentially expressed proteins was performed. As shown in Figure 5a, a number of vital pathways were affected by GCRV infection. The up-regulated acetylated proteins were only significantly enriched in ribosome pathway. However proteins with down-regulated acetylation levels were highly enriched in several pathways related to protein processing such as the protein processing in endoplasmic reticulum, biosynthesis of amino acids and protein export. The ribosome pathway enriched in the up-regulated proteins and the biosynthesis of amino acids enriched in the down-regulated proteins, together with the protein processing in endoplasmic reticulum suggested that the protein biosynthesis process may be widely modulated by viral infection. Besides, the down-regulated proteins were also enriched in cell metabolism process such as fructose and mannose metabolism, pentose phosphate pathway, galactose metabolism, glycolysis/gluconeogenesis, and carbon metabolism. Changes in the most significantly enriched pathway, protein processing in endoplasmic reticulum, are shown in Figure 5b. As indicated in this map, 10 proteins in this pathway were down-regulated at the lysine acetylation level including ERP57, GlcII, CRT, climp 63, UGGT, BiP, GRP94, PDIs, p97, Bap31. At the same time, there were still 6 proteins up-regulated including CNX, OSTs, NEF, heat shock protein 70 (Hsp70), Hsp90, Calpain. Taken together, these results indicate that the differentially expressed lysine acetylated proteins were highly associated with protein processing and metabolism.

### 2.6. Protein–Protein Interaction (PPI) Network Analysis of Differentially Expressed Proteins

**Figure 6:**
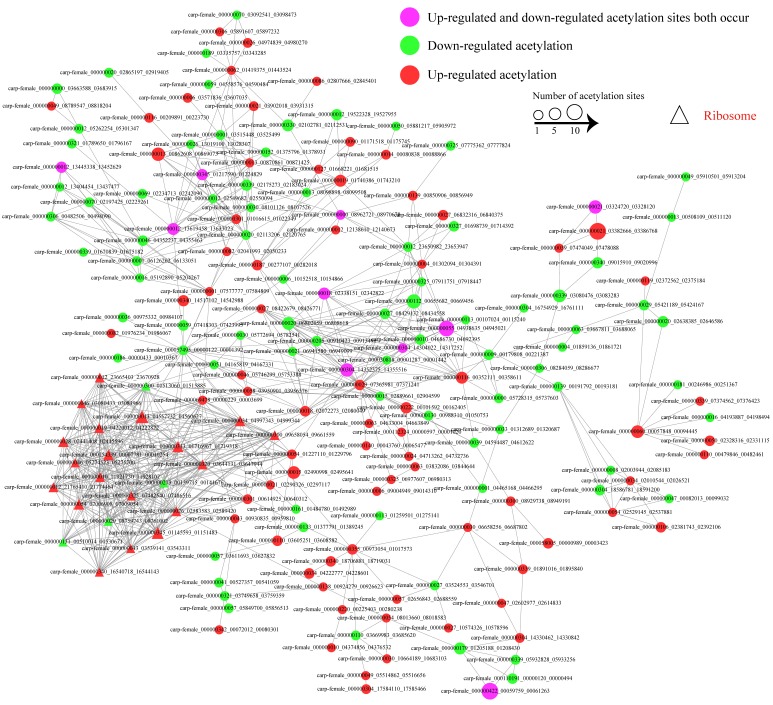
Interaction network of the quantified lysine acetylated proteins. The proteins associated with ribosome were shown in “△”. The proteins in green are down-regulated and the proteins in red are up-regulated. Proteins in purple were modified at different sites with both increased and decreased levels.

To further understand the influence of viral infection on the host protein interactions, we performed PPI network database search tool for the retrieval of interacting genes (STRING) combined with the complex detection algorithm MCODE. As there is no PPI data of CIK cell proteins in the STRING database, we used *Danio rerio* as the near-source species to get homologs proteins. Finally, 187 differentially expressed acetylated proteins were mapped to the protein network database. And there were as many as 687 PPIs among these proteins. The global network graph of these interactions was shown in Figure 6 and Table S2. As indicated in Figure 6, many proteins were involved in multiple interactions. Moreover, a tight protein–protein interaction network including 19 up-regulated proteins and 4 down-regulated proteins, the ribosomal protein network, was significantly enriched.

### 2.7. Suberoylanilide Hydroxamic Acid (SAHA) Treatment Inhibited the GCRV Infection, Whereas C646 Treatment Had No Obvious Effect on Virus Replication

Histone acetylation is critical for gene transcription and generally linked to many cellular processes, and the histone deacetylation is associated with transcriptional repression [29]. To understand whether there is a histone acetylation change happened in CIK cells upon viral infection, the related analysis was conducted. Six core histones with 16 lysine acetylation sites were found to be altered upon GCRV infection. Among which 7 lysine acetylation sites in 5 core histones were significantly altered in the acetylation level. Additionally, there were two lysine acetyltransferases up-regulated in the lysine acetylation level, MYST2 and Naa10. The sequences of the identified lysine acetylation peptides in histones and lysine acetyltransferases and the quantitative lysine acetylation profiles upon GCRV infection are summarized in Table 1. These results confirmed that the histone acetylation profile was modified upon GCRV infection.

**Figure 7:**
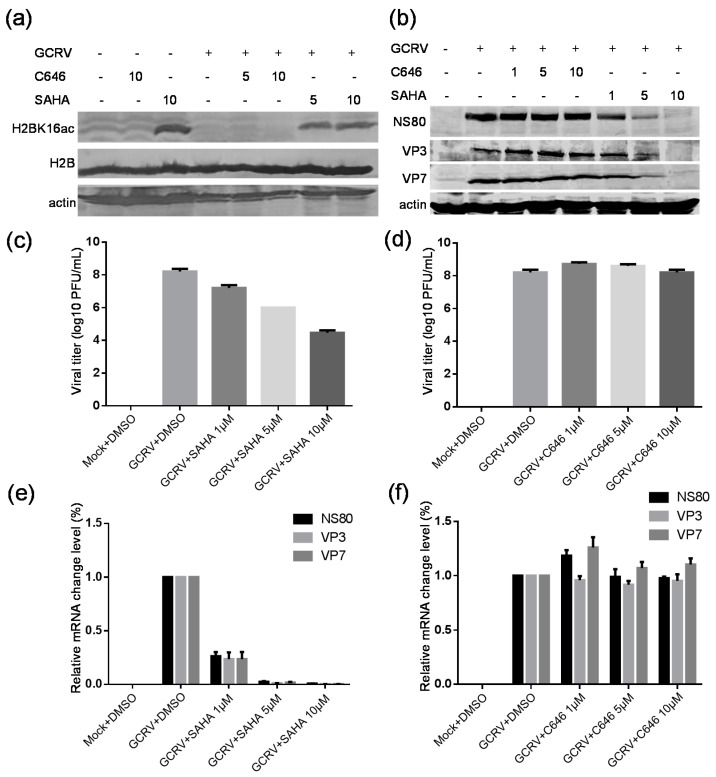
The effect of SAHA or C646 treatment on GCRV infection in CIK cells. (**a**) The influence of C646 and SAHA on H2BK16 acetylation. Cells were infected or mock infected with GCRV at a multiplicity of infection (MOI) of 1 in the presence or absence of SAHA or C646 at the indicated concentrations. Then cells were collected at 18 h post-infection and subjected to Western blotting analysis by the indicated antibodies; (**b**) Cells were infected with GCRV at an MOI of 1 in the presence or absence of SAHA or C646 at the indicated concentrations. Then cells were collected at 18 h post-infection and subjected to Western blotting analysis for the expression of NS80, VP3 and VP7 by the indicated antibodies; (**c**–**f**) Cells were infected with GCRV at an MOI of 1 in the presence of SAHA (**c**,**e**) or C646 (**d**,**f**). Then the cultured cell supernatants or cells were collected respectively at 18 h post-infection (h.p.i.) and subjected to viral plaque assay (**c**,**d**) or quantitative RT-PCR for NS80, VP3 and VP7 transcript level analysis (**e**,**f**). Data shown are mean ± standard deviation of three individual experiments.

SAHA is a well-known pan histone deacetylase inhibitor, and can significantly increase the acetylation level of most histone lysine sites [30]. C646 is a competitive and selective inhibitor of p300/CBP histone acetyltransferase, and can impede intracellular histone acetylation in several cell lines [31]. Some previous reports have shown that SAHA had antiviral effects by suppressing HCV and RSV replication [21,22], and C646 has been found to inhibit the influenza virus replication [23]. To determine the effect of the drugs on host cells in response to GCRV infection, biochemical assays were further performed with histone lysine acetylation sequence-specific antibodies. As shown in Figure 7a, SAHA treatment significantly increased the acetylation level of host cells in H2BK16 with or without viral infection, while C646 treatment did not obviously affect the acetylation level. Subsequently, the effects of SAHA or C646 treatment on viral infection and replication were also investigated using antibodies against viral nonstructural protein NS80 and capsid proteins VP3 and VP7. As shown in Figure 7b, the expression levels of three viral proteins were down-regulated gradually following the increased concentration of SAHA, while no evident changes were detected with C646 treatment even when the concentration of C646 was reached 10 µM. To confirm the above data, the viral titers towards SAHA or C646 treatment were tested. Results showed that SAHA treatment decreased the viral titers approximately 4 logs by comparing to positive control, but no significant effect on the viral replication with C646 treatment was detected (Figure 7c,d). To further prove whether C646 or SAHA affects the viral protein expression or viral replication through gene transcription, quantitative real-time PCR analysis was carried out. As shown in Figure 7e,f, the relative mRNA levels of GCRV proteins NS80, VP3 and VP7 obviously reduced with 1 µM of SAHA treatment and decreased more with the increased concentration, whereas the mRNA levels were not distinctly changed by the C646 treatment. All together, these data indicated that SAHA treatment can inhibit the viral infection in a dose-dependent manner, but C646 treatment cannot significantly affect the viral replication.

## 3. Discussion

Although protein lysine acetylation is an important and highly conserved PTM with diverse biological functions in both eukaryotes and prokaryotes, little is known about this modification in aquatic species. Here, we performed a genome-wide protein acetylation analysis to elucidate the effects of GCRV infection on the protein lysine acetylation profiles of CIK cells in order to gain insight into GCRV pathogenesis. To our knowledge, this is the first systematic analysis of lysine acetylated proteins in response to aquareovirus infection covering the entire acetylome of fish cells.

Totally, 1391 lysine acetylation sites in 832 proteins were identified in viral infected cells compared to control cells. These acetylated proteins had a broad range of biological functions and were involved in diverse cellular processes. Most of these proteins were modified at single lysine acetylation site. However, some proteins were changed at multiple sites including KRT8 (12 sites), KRT18 (7 sites), plectin (14 sites) and PDIA3 (9 sites). KRTs are intermediate filament proteins which act as essential cytoskeletal component involved in the maintenance of cell morphology [32,33]. Plectin binds to various cytoskeletal proteins including microtubules and intermediate filaments and regulates cellular survival, growth, and polarization signaling pathways, and also can modulate the mechanical properties of keratin K8/K18 networks [34,35]. PDIA3 is a thiol-oxidoreductase chaperone with multiple functions, including the proper folding of newly synthesized proteins and assembly of major histocompatibility complex I [36]. These results suggested that GCRV infection induced wide changes in host cellular proteins at lysine acetylation level. Additionally, many lysine acetylation motifs identified in this study were also found in other eukaryotic and prokaryotic species [37,38,39,40,41,42], indicating that the lysine acetylation motifs are highly conserved among different species.

GCRV is a fusogenic aquareovirus which replicates efficiently in fish CIK cells. Its infection can induce cell-cell membrane fusion between virus-infected cells and neighboring uninfected cells and form large syncytia to facilitate viral spreading [3,6]. Since these processes involve the plasma membrane, changes in membrane proteins and rearrangement of cell structures should be accompanied with the viral infection. In this study, many up- or down-regulated plasma membrane proteins were found based on subcellular localization analysis and GO functional classification analysis, suggesting plasma membrane proteins acetylation was widely altered at the lysine acetylation level by viral infection. In addition, we found that proteins related to myosin complex and gelsolin were highly enriched based on GO clustering result and protein domain analysis. The actin-dependent molecular motor myosins interact with actin filaments and are responsible for a wide range of motility processes [43,44]. Gelsolin is an actin-modulating protein that binds to the barbed ends of actin filaments [45]. In this regard, it is possible that the increase of the acetylation level in myosins might be helpful for cell–cell fusion process that is required for efficient nascent aquareovirus infection. These data also suggested that the GCRV infection changed the acetylation of proteins involved in motility processes, which may be related to the syncytia formation in cells caused by GCRV infection.

Notably, proteins related to homeostasis, including Hsp90, PDIA3, PDIA4 and so on, were also highly enriched in Q1 with down-regulated lysine acetylation sites (Table S1). Hsp90, which was considered to be very important for protein homeostasis [46], was found to have 5 lysine acetylation sites significantly down-regulated by GCRV infection (Table S1). Hsp90 is regulated by the dynamics of acetylation and deacetylation, thus affecting the selection of chaperones and clients [47]. Moreover, Hsp90 is a highly conserved molecular chaperone that regulates diverse cellular processes through interaction with client proteins. The fact that 5 down-regulated lysine acetylation sites of Hsp90 was identified in this study suggested that widespread processes were regulated and the cellular homeostasis might be broken down due to efficient viral replication. This may also be related to the typical hemorrhagic symptom of diseased fish caused by productive viral infection. Histones are the chief protein components of chromatin and play key role in gene regulation. Glycoside hydrolase superfamily and acyl-CoA-binding proteins are involved in metabolism. Guanylate-binding proteins are induced by interferon-gamma and are important to the protective immunity against microbial and viral pathogens [48]. CDC48 functions in a broad range of cellular processes and can regulate the ubiquitin related events [49]. We found from protein domain analysis that translation proteins, histone core, histone-fold, glycoside hydrolase superfamily, CDC48 domain 2, CDC48 domain 2-like, guanylate-binding protein (C-terminal), acyl-CoA-binding protein, and so on were highly enriched in host cells upon GCRV infection. These data suggested that extensive intracellular activities were changed by GCRV infection.

PPI network of the up- or down-regulated proteins was also established. A variety of interactions in host cells were found to be modulated upon viral infection at the acetylation level. Among these interactions, it was observed that the differentially expressed acetylated proteins were highly enriched in ribosome (23 proteins), which were mainly up-regulated at the lysine acetylation level. This is consistent with the KEGG pathway result that the ribosome pathway is significantly enriched in the up-regulated proteins. In support of our observation, the ribosome protein interaction network was also enriched in the acetylome analysis of human borna disease virus infected cells [24], suggesting that the ribosome pathway was greatly changed in response to GCRV infection. In consistent with this phenomenon, the nonstructural protein σNS of mammalian orthoreoviruses, homologous protein of GCRV nonstructural protein NS38, strongly interacted with eIF3A and pS6R in the 43S pre-initiation complex, hinting that σNS recruits or maintains ribosomes within viral factories to facilitate viral translation [50]. Also, many proteins in the PPI network were involved in multifarious protein–protein interactions, such as Hsp90ab1 (carp-female_000000029_07365981_07371241), Hsp90b1 (carp-female_000000020_06802059_06808618), Hspa8 (Hsp70, carp-female_000000304_14352375_14355516), eukaryotic elongation factor (eEF) eEF1a1l1 (carp-female_000000054_01227110_01229796), eEF2b (carp-female_000069478_00000229_00003699), calnexin (canx) (carp-female_000000116_00352111_00358611), atp5c1 (carp-female_000000019_01740386_01743210), atp5a1 (carp-female_000000187_00277107_00282018) and so on (Figure 6 and Table S2). Hsps are important evolutionarily conserved proteins that are present in all organisms. Except for their molecular chaperone functions, Hsps are also involved in diverse cellular processes, such as protein metabolism and homeostasis, immune defense reactions, signal transduction and so on [51,52,53]. The alterations on the interaction of Hsps with other proteins are consistent with the GO enrichment result and the KEGG pathway result, suggesting that widespread processes especially the cellular homeostasis and metabolism were comprehensively altered in response to viral infection. The eEF-1 and eEF-2 mediate the peptide chain elongation in eukaryotes. The eEF-1 mediates the binding of cognate aminoacyl-tRNA to the ribosome and eEF-2 catalyses the movement of the ribosome relative to the mRNA during the peptide elongation stage of protein synthesis [54,55]. This findings are consistent with the KEGG pathway results that the ribosome pathway, the biosynthesis of amino acids and the protein processing in endoplasmic reticulum were highly enriched, suggesting that the protein biosynthesis process was widely modulated by viral infection. Canx is an abundant calcium binding chaperone in endoplasmic reticulum, which is essential in the correct folding of membrane proteins of eukaryotic cells. Canx also regulates ER stress-mediated apoptosis in a manner independent of its chaperone functions [56,57]. It is possible that GCRV might utilize the canx to damage the regularly operated cellular membrane system and thus facilitate the cell–cell membrane fusion process during infection. Although direct functional data are lacking, these results hint that a wide range of protein interactions were modulated toward GCRV infection at the lysine acetylation level.

Protein lysine acetylation is important for regulating cellular metabolism [16,58]. Here, a large group of acetylated proteins altered by GCRV infection are found to be involved in metabolism based on GO classification analysis. The KEGG pathway enrichment analysis also indicated that down-regulated proteins were significantly enriched in glycolysis/gluconeogenesis, fructose and mannose metabolism, pentose phosphate pathway, galactose metabolism, and carbon metabolism. Four of the significantly enriched pathways were related to carbohydrate metabolism including glycolysis/gluconeogenesis, galactose metabolism, pentose phosphate pathway and fructose and mannose metabolism. These data suggest that the cellular metabolism was greatly altered due to GCRV infection.

Lysine acetyltransferases are able to acetylate specific lysine residues within histones or other nonhistone proteins. In this study, two lysine acetyltransferases were also found to be up-regulated in the lysine acetylation level, MYST2 and Naa10 (Table S1). MYST2, also known as KAT7, has been confirmed to have acetyltransferase activity and its deficiency could lead to almost complete loss of global histone-H3 lysine 14 acetylation [59]. The human Naa10 has been linked to multiple functions, including N-terminal acetyltransferase, lysine acetylation, and acetyltransferase-independent functions [60]. Similar to human Naa10, the Naa10 in zebrafish also has N-terminal acetyltransferase activity and is essential for normal development and viability of zebrafish [61]. The role of hyperacetylation on Naa10 and MYST2 is unknown, and there has been no previous link between these proteins and viral infection. However, the altered acetylation of some proteins in response to infection may be due in part to the hyperacetylation of these acetyltransferases. It is reasonable to speculate that the altered acetylation of these acetyltransferases may lead to the comprehensive changes in amounts of host proteins identified in this study.

Several previous studies have reported virus induced changes in histone lysine acetylation sites, such as adenovirus [62], borna disease virus [25,63], including decreased and increased acetylation. In this study, 16 histone lysine acetylation sites were identified and quantified, of which five sites were significantly down-regulated and two sites were significantly up-regulated (Table 1), suggesting the histone acetylation profile was altered in response to GCRV infection. This result also hints that the HDACs and HATs might perform certain functions in CIK cells upon viral infection. Based on previous reports that the HDAC inhibitor SAHA can suppress HCV and RSV replication and the HAT inhibitor C646 can inhibit the influenza virus replication [21,22,23], we further investigated the effects of SAHA and C646 in GCRV replication. By analyzing the viral titers and protein expression or mRNA levels in CIK cells after incubating virus with either SAHA or C646, we found that C646 treatment did not obviously affect the viral infectivity, whereas SAHA treatment markedly reduced the viral replication, suggesting a possible role of SAHA on GCRV replication. This result is similar to the previous reports of SAHA on HCV and RSV replication. Considering that SAHA is a pan histone deacetylase inhibitor, it is needed to further make an intensive investigation to reveal the natural molecular mechanisms underlying the suppressive effect of SAHA on GCRV replication.

## 4. Materials and Methods

### 4.1. Cell, Virus and Antibodies

The CIK cell line was maintained in authors’ laboratory, and grown in minimal essential medium (MEM; Gibco-BRL, San Francisco, CA, USA) supplemented with 10% fetal bovine serum (FBS; Gibco-BRL) at 28 °C. GCRV-873, the type strain of grass carp reovirus isolated and stored in the authors’ laboratory as described previously [3,4], was used in this experiment. The polyclonal antibodies against GCRV core protein VP3, outer capsid protein VP7 and nonstructural protein NS80 were generated in authors’ laboratory [64]. Mouse monoclonal antibody against β-actin was purchased from Santa Cruz Biotechnology (Santa Cruz, CA, USA). Pan anti-acetylation antibody, acetyl-Histone H2B and acetyl-Histone H2B (Lys16) mouse monoclonal antibody were the products of PTM. Biolab Hangzhou (Hangzhou, China).

### 4.2. Viral Infection

GCRV was propagated in the CIK cell monolayer in 50 cm^2^ flasks (Corning Inc., Corning, NY, USA) at a concentration of 2 × 10^6^ cells/ml as previously reported [65]. Briefly, when cells were grown to 80% confluence, the monolayers were infected with GCRV at a MOI of 1. Meanwhie, the mock-infected cells were treated with same amount of medium in the same conditions. When cytopathic effects were observed at 18 h.p.i., the infected cells and non-infected cells were prepared and harvested for further proteomic analyses. Two rounds of independent experiments were performed.

### 4.3. Protein Extraction and Western Blotting Analysis

The collected virus infected cell cultures were centrifuged with 12,000 rpm for 5 min at 4 °C and washed twice with cold PBS. Then, each sample with about 1 × 10^8^ total cells were sonicated three times on ice in lysis buffer (8 M urea, 10 mM DTT and 1% Protease Inhibitor Cocktail III (Merck Millipore, Billerica, MA, USA), 2 mM EDTA, 3 µM TSA, 50 mM NAM) by using high intensity ultrasonic processor (Scientz, Ningbo, China) at 270 W for five min. The remaining debris was removed by centrifugation at 12,000× *g* at 4 °C for 10 min. The protein concentration was determined with 2-D Quant kit according to the manufacturer’s instructions. Western blotting analysis was performed as previously described [6]. Briefly, whole-cell extracts with equal amounts of 10 μg were subjected to 10% SDS-PAGE and transferred to a polyvinylidene fluoride (PVDF) membranes, followed by blocking with 5% nonfat milk in Tris-buffered saline-Tween (TBST) and probed with primary antibodies (pan anti-acetylation antibody (1:1000), mouse monoclonal antibody against β-actin (1:2000), polyclonal antibodies against GCRV core protein VP3 (1:500), outer capsid protein VP7 (1:500) and nonstructural protein NS80 (1:500), acetyl-Histone H2B and acetyl-Histone H2B (Lys16) mouse monoclonal antibodies (1:2000)) at 37 °C for 2 h. After washing with TBST, the membrane was incubated with secondary antibody conjugated with alkaline phosphatase (1:1000). Specific protein bands were developed with AP substrate solution (NBT/BCIP).

### 4.4. Trypsin Digestion, Dimethylation Labeling and Affinity Enrichment

The proteins were digested with trypsin as previously described [37]. In detail, the protein sample was diluted after adding 100 mM NH_4_CO_3_ to a urea concentration of less than 2 M. Then, trypsin (Promega, Madison, CT, USA) was added at 1:50 trypsin-to-protein mass ratio for the first digestion at 37 °C overnight and a 1:100 trypsin-to-protein mass ratio for a second four-hour digestion. After trypsin digestion, peptide was desalted by Strata X C18 SPE column (Phenomenex, Torrance, CA, USA) and vacuum-dried. Peptide was reconstituted in 0.1 M NaAc (pH = 5.99). The samples are differentially isotope labeled in parallel in different tubes by adding four µL CH_2_O or CD_2_O to the samples to be labeled with light (control Cell) and heavy (viral infected cell) dimethyl, respectively. The reactions were mixed briefly and added four µL of 0.6 M NaBH_3_CN, incubated in a fume hood for one hour at room temperature. Finally, the labeling reactions were quenched by adding 16 µL of 1% ammonia solution, then adding formic acid for desalted and dried by vacuum centrifugation. Then the tryptic peptides were subjected to lysine acetylated peptide enrichment using a previously described method [66]. The resulting peptides were cleaned with C18 ZipTips (Merck Millipore) according to the manufacturer’s instructions, followed by LC–MS/MS analysis.

### 4.5. LC–MS/MS Analysis

For LC–MS/MS analysis, peptide samples were dissolved in 0.1% formic acid, directly loaded onto a reversed-phase pre-column (Acclaim PepMap 100, Thermo Fisher Scientific, Waltham, MA, USA). As usual, we used reversed-phase analytical column (Acclaim PepMap RSLC, Thermo Fisher Scientific) for peptide separation as previously reported [66]. The resulting peptides were analyzed by Q Exactive™ Plus hybrid quadrupole-Orbitrap mass spectrometer (Thermo Fisher Scientific). Intact peptides were detected in the Orbitrap and all the Peptides were selected for MS/MS using NCE by setting as 28. The ion fragments were detected in the Orbitrap at a resolution of 17,500. Automatic gain control was used to prevent overfilling of the ion trap and 5E4 ions were accumulated for generation of MS/MS spectra. For MS scans, the *m*/*z* scan range was set as 350 to 1800.

### 4.6. Database Search

The protein acetylation site identification and quantification were processed using MaxQuant with integrated Andromeda search engine (v. 1.4.1.2). Tandem mass spectra were searched against *Ctenopharyngodon idellus* database (32,828 sequences of “C_idella_female_genemodels.v1.aa.gz” on http://www.ncgr.ac.cn/grasscarp/) concatenated with reverse decoy database. Trypsin/P was specified as cleavage enzyme allowing up to four missing cleavages, five modifications per peptide and five charges. DimethLys0, DimethNter0 were specified as a light label group and DimethLys4, DimethNter4 were specified as heavy group. Mass error was set to five ppm for main search and 0.02 Da for fragment ions. The other parameters in MaxQuant are set as previously described [66]. The lysine acetylation site localization probability was set as >0.75.

### 4.7. Bioinformatics Analysis

The identified acetylated proteins were analyzed using motif-x to characterize the model of sequences constituted with amino acids in specific positions of modifier-21-mers (10 amino acids upstream and downstream of the site). The subcellular localization was predicted using an updated version of PSORT/PSORT II, the WoLF PSORT program. GO annotation proteome was derived from the UniProt-GOA database (http://www.ebi.ac.uk/GOA/). Then proteins were classified based on three categories: biological process, cellular component and molecular function. Domain annotation was performed using the InterProScan based on protein sequence alignment method, and the InterPro domain database was used. KEGG database was used to annotate protein pathways. GO, protein domain and KEGG pathway enrichment analysis were performed using the DAVID bioinformatics resources 6.7. Enrichment-based clustering analysis was performed as previously described [67]. The Cluster membership was visualized by a heat map using the “heatmap.2” function from the “gplots” R-package. The STRING database system (http://string-db.org/) version 10.0 was used to get the protein interaction network. All interactions that had a confidence score ≥0.7 (high confidence) were fetched. The Interaction network form STRING was visualized in Cytoscape (version 3.0).

### 4.8. Pharmacological Inhibitors and Cell Viability Assay

Inhibitors SAHA and C646 were purchased from Sigma-Aldrich (St. Louis, MO, USA). CIK cells were seeded in 96-well plates and grown overnight at 28 °C, then incubated with different concentrations of the drug SAHA or C646 for 18 h. Cell viability was examined with Cell Counting Kit-8 (CCK-8, Beyotime Institute, Haimen, China) according to the manufacturer’s instruction. The optical density was determined at 450 nm using a microplate reader (Bio Tek, Winooski, VT, USA).

### 4.9. Analysis of Viral Growth after Treatment with Drugs

To analyze the effect of histone acetylation change of CIK cells treated with drugs on GCRV infection, the cells were treated with 1, 5, 10 µM inhibitor SAHA or C646 for 1.5 h and then infected with GCRV at an MOI of one. After one h of adsorption at 28 °C, the infected cells were washed with PBS and then added fresh MEM-2 (with 2% FBS) containing inhibitors. Infected cells were incubated at 28 °C for 18 h cultivation. The expression levels of cell histone acetylation or viral protein were analyzed by Western blotting. Viral yields were determined by plaque assay on CIK cells as previously described [8].

### 4.10. Real-Time Quantitative PCR Analysis

The virus genome was amplified by real-time quantitative PCR for detecting the expressions of viral protein VP3, VP7 and NS80 as described elsewhere [64]. In details, total RNAs were firstly extracted using Simply P Total RNA Extration Kit (BIOER Hangzhou, Hangzhou, China) according to the manual instruction from infected CIK cells at 18 h.p.i. Next, first-strand cDNA was synthesized by adding one µg RNA template and reverse primer in a total volume of 20 µL. Then quantitative PCR was performed on a real-time thermo cycler (Bio-Rad, Hercules, CA, USA) with the CFX 96 software. All the primers used in this study were determined by the slope value of the standard curve, and displayed amplification efficiency of greater than 90% as reported previously [64]. The iTaq Universal SYBR Green Supermix (Bio-Rad) was used to quantify expression. Amplification was performed by the following parameters: denaturing at 95 °C for three min, followed by 40 cycles of 95 °C for 15 s and 60 °C for 30 s. Each reaction was performed in triplicate and β-actin was analyzed as internal control for each experiment. Each assay was performed three times independently. The relative expression ratios were calculated by using a mathematical model and Graphpad Prism V5.01 was used for relative quantification.

### 4.11. Statistical Analysis

All graphs represent means and standard deviations of normalized data points for triplicate samples from each of representative experiments. Mean values were compared using Student’s *t* test (GraphPad Prism 6, GraphPad Software, Inc., San Diego, CA, USA), *p* values of 0.05 were considered to be statistically significant.

## 5. Conclusions

In conclusion, combining with quantitative proteomic approach and bioinformatics analysis, we determined the effects of GCRV infection on the acetylome profiles of CIK cells by using a most pathogenic aquareovirus strain GCRV-873, and demonstrated that aquareovirus infection comprehensively impacted host acetylome. The acetylated proteins altered by GCRV infection were distributed in a wide range of cellular compartments and linked to diverse biological functions. Moreover, biological experiments indicated that the HDAC inhibitor SAHA could significantly suppress the GCRV infection. To our knowledge, this is the first systematical description of acetylated proteins in response to aquareovirus infection covering the entire acetylome of fish cell line. This study provides a strong evidence to further investigate the crosstalk between ubiquitination and phosphorylation for elucidating the biological functions of host cell PTM changes in piscine model upon viral infection.

## Figures and Tables

**Table 1:** Summary of quantifiable changes of lysine acetylation sites in histones and lysine acetyltransferases of CIK cells in response to GCRV infection.

Histone and Modified Sites	Modified Sequence	Normalized Ratio H/L (↑↓) ^1^
H1-βK168ac	K(ac)YPSVEMDK	0.572 **↓**
H1.0K43ac	ATSHPK(ac)YSEMIK	1.049
H1.0K68ac	QSIQK(ac)YVK	1.027
H1.0K75ac	NHYK(ac)VGDNADSQIK	1.272
H2B3K16ac	K(ac)AVTK(ac)TQK	2.215 **↑**
H2B3K20ac	K(ac)AVTK(ac)TQK	2.215 **↑**
H2BK19ac	VTK(ac)TAGK(ac)SGK	0.658 **↓**
H2BK23ac	VTK(ac)TAGK(ac)SGK	0.678
H2BK115ac	HAVSEGTK(ac)AVTK(ac)YTSSK	0.789
H2A.xK130ac	TGQAVASSGK(ac)SGK(ac)K	0.307 **↓**
H2A.xK133ac	TGQAVASSGK(ac)SGK(ac)K	1.232
H4K19	K(ac)QLATK(ac)AAR	0.617 **↓**
H4K24	QLATK(ac)AAR	0.539 **↓**
H4K57	YQK(ac)STELLIR	0.89
H4K80	EIAQDFK(ac)TDLR	0.808
H4K123	VTIMPK(ac)DIQLAR	0.826
MYST2K137	CPTPGCNSLGHLTGK(ac)HER	1.514 **↑**
Naa10K101	FQISEVEPK(ac)YYADGEDAYAMK	2.365 **↑**

^1^ The up-regulated proteins were indicated by “**↑**” and down-regulated proteins were indicated by “**↓**”.

## Supplementary Materials

Supplementary materials can be found at www.mdpi.com/1422-0067/18/11/2419/s1.

## Abbreviations

GCRV	Grass carp reovirus
CIK	*Ctenopharyngodon idella* kidney
HDAC	Histone deacetylase
SAHA	Suberoylanilide hydroxamic acid
PTM	Post-translational modification
HAT	Histone acetyltransferase
HIV	Human immunodeficiency virus
HPV	Human papilloma virus
RSV	Respiratory syncytial virus
HCV	Hepatitis C virus
KRT	Keratin
PDIA3	Protein disulfide isomerase family A member 3
GO	Gene ontology
PCI domain	Proteasome component domain
KEGG	Kyoto encyclopedia of genes and genomes
PPI	Protein–protein interaction
STRING	Search tool for the retrieval of interacting genes
MOI	Multiplicity of infection
Hsps	Heat shock proteins
eEF	Eukaryotic elongation factor
canx	Calnexin
MEM	Minimal essential medium
FBS	Fetal bovine serum
h.p.i.	Hours post-infection
PVDF	Polyvinylidene fluoride
TBST	Tris-buffered saline-tween
