# Supplementary figures and images for: Comparative Proteomic Analysis of Lysine Acetylation in Fish CIK Cells Infected with Aquareovirus
Source: Int J Mol Sci. 2017 Nov 14;18(11):2419. doi: 10.3390/ijms18112419 (PMC5713387; doi:10.3390/ijms18112419)

(a)

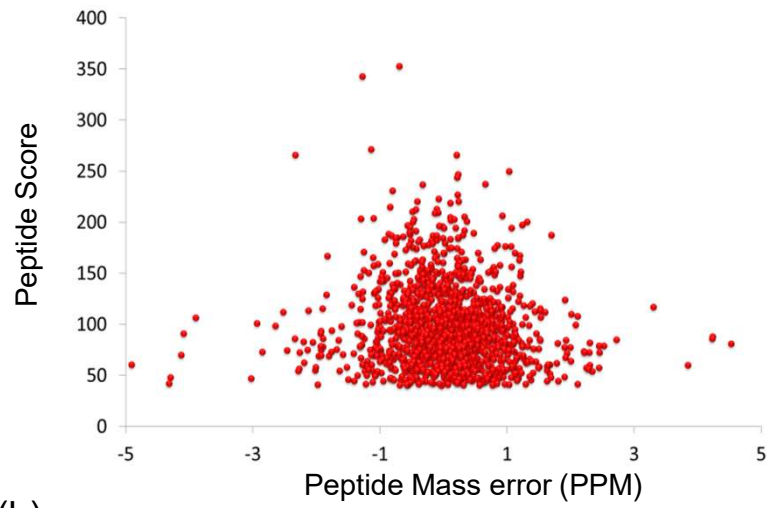

(b)

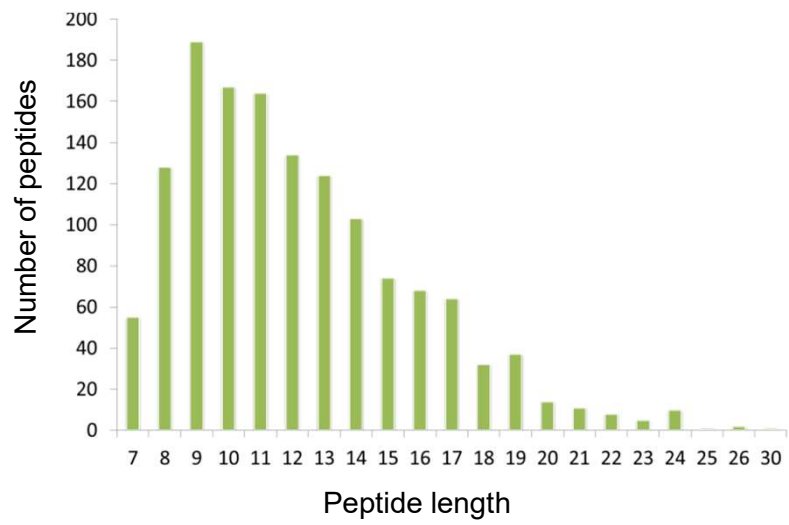

Supplement: Supplementary file 1 [file ijms-18-02419-s001.zip › Supplementary materials/Fig-S1.pdf]
